# A Method to Calibrate the Carbon Dioxide (Chemical) Stimuli of Pneumatic Esthesiometer Externally

**DOI:** 10.1167/tvst.8.5.4

**Published:** 2019-09-11

**Authors:** Varadharajan Jayakumar, Trefford L. Simpson

**Affiliations:** 1 School of Optometry and Vision Science, University of Waterloo, Waterloo, Ontario

**Keywords:** pneumatic esthesiometer, calibration, psychophysics, corneal sensitivity, carbon dioxide

## Abstract

**Purpose:**

To determine the feasibility of using a portable carbon dioxide (CO_2_) sensor to calibrate a pneumatic esthesiometer and then to calibrate the chemical stimuli.

**Methods:**

The chemical stimuli in ocular surface experiments are combinations of medical air and added CO_2_ (%CO_2_). These stimuli were calibrated using a portable CO_2_ sensor (COZIR CM-0041) and data logger, delivered for 100 seconds by using the Waterloo Belmonte esthesiometer. The distances between the sensor and esthesiometer tip were 0 (to measure feasibility), 3, 5, and 10 mm. In experiment I, 100% CO_2_ was tested using four different flow rates (50, 100, 150, and 200 mL/min) at three working distances. In experiment II, flow rates of 20 to 100 mL/min and concentrations of 20% to 100% CO_2_ were tested in 20 steps at 3 working distances.

**Results:**

The CO_2_ sensor correctly reported the esthesiometer extremes of 0% and 100% CO_2_ when placed at the esthesiometer tip. There were progressive, systematic increases in concentrations reaching/reported by the sensor with increasing flow rates and nominal concentrations and progressive decreases in measurements with increases in working distance.

**Conclusions:**

CO_2_ concentrations in pneumatic esthesiometers can be calibrated and, as expected, vary with flow rate and distance, highlighting the importance of calibration and standardization of CO_2_ stimuli in these instruments.

**Translational Relevance:**

Calibrated CO_2_, a chemical sensory stimulus in humans, may be used in testing the surface of the eye as well as other membranes within which the CO_2_ can be dissolved (e.g., mucous) to produce an acidic stimulus.

## Introduction

Esthesiometers have been used in estimating the sensitivity of various sensory systems, such as skin, particularly for measuring the touch sensitivity/pain sensitivity, each of which depends on the amount of pressure applied on the surface of interest.^1^ Like other sensory systems, the sensitivity of the ocular surface has also been measured using esthesiometers.^2^–^11^ Von Frey^12^ developed the horsehair-based esthesiometer to measure the mechanical sensitivity of the ocular surface.^13^ The esthesiometer's filaments are of a certain length and diameter to exert a precalibrated amount of pressure on the ocular surface.^12^ The fundamental principle of stimulating mechanical sensitivity with a filament proposed by Von Frey was widely accepted, and many versions of the esthesiometers were developed to quantify corneal sensitivity; in ophthalmic research, perhaps the most significant of them is the Cochet-Bonnet (CB) esthesiometer that is used clinically as well as in research settings.^2^,^4^,^6^,^14^,^15^ However, the filament stimuli are unidimensional, as they measure the mechanical sensitivity of a localized area with a narrow dynamic range of stimulus intensity. Other limitations that have been documented include perceptible filament producing an anxious response when brought closer to the eyes^16^ and a variable/inconsistent pressure being applied to the ocular surface due to the bending of the filament.^3^,^17^,^18^ Even though a number of devices were developed with the limitations of the CB esthesiometer addressed,^3^,^7^,^14^,^15^,^19^ clinically, the CB esthesiometer is still the most frequently used esthesiometer. Other esthesiometers have been developed to measure corneal sensitivity, including Lele and Weddell's^3^ infrared heated air stimulus, Schirmer's^14^ esthesiometer with a broader contact surface, Larson's^15^ electromechanical esthesiometer, and Tanelian and Beuerman's^7^ heated saline jet, and there has been a report of a CO_2_ laser ocular surface esthesiometer.^19^

Based on the reports of the cutaneous polymodal nociceptor's responsiveness to chemical stimuli, such as acetic acid and capsaicin, Belmonte's group^8^,^20^–^24^ recorded the single unit electrical activity of cat and rabbit corneas by using the same chemical stimuli and developed a pneumatic esthesiometer for human participants. CO_2_ has been identified as an ideal stimulus for the human ocular surface chemical sensitivity experiments because of the sustained reduction in the pH of the ocular surface, unlike a buffered response obtained by an acetic acid stimulus.^9^ In a number of studies, corneal chemoreception using CO_2_ was measured and illustrated, perhaps, the importance of measuring chemical sensitivity.^9^,^24^–^29^ What emerged over a series of corneal physiological and psychophysical experiments was the demonstration of the utility of a pneumatic esthesiometer capable of measuring responses to mechanical, chemical, and thermal stimulation, and linking hypotheses were developed and empirically supported that in humans there are channels with similar attributes to the neural behavior reported in rabbit and cat corneas.^9^,^24^,^25^,^30^,^31^ Also, the experiments demonstrated that in animals (mainly cat and rabbit, initially), polymodal nociceptors were found to form a majority (about 70%) of corneal receptors, with mechanonociceptors (20%) and cold receptors (10%) forming the remaining corneal receptor population.^32^ It was hypothesized that these polymodal subgroups form the main peripheral sensory input from the cornea for the detection of nociceptive chemical, thermal, and mechanical stimuli.^1^,^32^,^33^

There are different versions of pneumatic esthesiometers described in the literature,^10^,^24^,^25^,^27^,^34^–^37^ all of which were custom built or modified versions of Belmonte's design that delivers air/CO_2_ to the ocular surface. There are few reports on the calibration of the flow rate and temperature of the pneumatic stimulus.^25^,^38^–^40^ However, and perhaps because of technical issues, there are no reports on the calibration of CO_2_ stimulus of the pneumatic esthesiometer.^41^,^42^ The CO_2_ is controlled and calibrated internally either in the control box where the gas mixing occurs or at the nozzle with a closed-loop tube sampling CO_2_ sensors.^24^,^25^ Even though the CO_2_ is internally calibrated, the %CO_2_ in the stimulus is unknown/not calibrated when it reaches the ocular surface (the place at which the pneumatic stimulus actually operates). The gases are not restricted to a closed column and so the stimulus has to interact with the air in the environment between the nozzle and the ocular surface. The physical chemistry at the level of ocular surface will be different from the tip of the esthesiometer, but it is unclear how much of the CO_2_ is retained by the possible laminar flow (or otherwise) within the stimulus column.^10^

Previously, the only way to measure %CO_2_ in the stimulus externally was to use solid electrolyte sensors that were typically difficult to use and have long and short term drift effects, making the measurements less reliable over time.^43^ Recent advancements in the CO_2_ sensors have made them more reliable to measure at ambient conditions and offer wider concentration detection range. These are solid-state nondispersive infrared (NDIR) sensors that are portable sensors that use a low-power infrared light-emitting diode and detector to estimate the CO_2_ levels.^43^ Because these sensors have not been used previously for the calibration of esthesiometer stimulus, in this work, we initially determined the feasibility of using the sensor for calibration of the esthesiometer stimulus and then calibrated the CO_2_ stimuli at different concentrations, flow rates, and working distances.

## Materials and Methods

### Waterloo Modified Belmonte Esthesiometer

**Figure 1:**
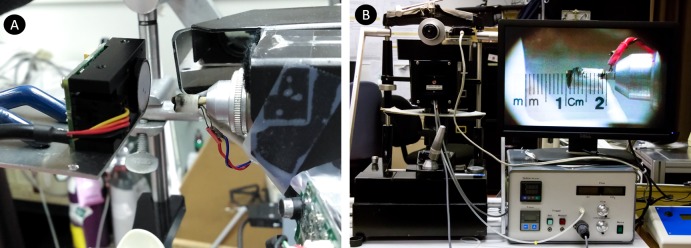
(A) Setup of modified Belmonte esthesiometer and COZIR CM-0041 CO_2_ sensor; and (B) the esthesiometer setup with the control box and calibrated viewing system.

**Figure 2:**
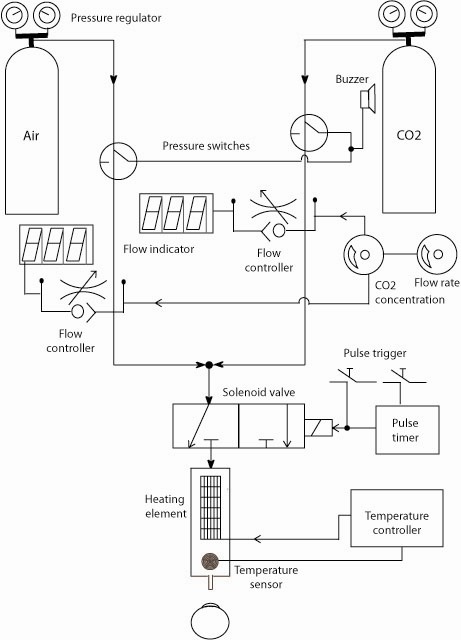
The Waterloo modified Belmonte esthesiometer (adapted from the thesis of Situ^44^).

The construction of the Belmonte esthesiometer has been discussed in detail by Belmonte et al.^24^ The essential components of an esthesiometer are the gas inputs, control box, and nozzle. The gas inputs to the control box are regulated at 5 psi from both the medical air and carbon dioxide (CO_2_) cylinders. The control box houses the electronic controls for manual input and flow meters/gas mixers to prepare the stimulus. Our esthesiometer (Waterloo version) has been extensively modified to include automation of flow control, mixing, and stimulus delivery (as well as the audio prompts and subject data collection) (Figs. 1 and 2).^44^ The stimulus is delivered through the nozzle mounted on an adjustable mount, controlling the x, y, and z position and yaw. The tip/nozzle of the esthesiometer was wrapped with a coil thermostat to control the temperature of the stimulus delivered (Figs. 1 and 2). A calibrated camera viewing system mounted on the side of the esthesiometer allows the examiner to position the tip at the desired working distance and partly control/monitor the stimulus orthogonality relative to the ocular surface. To create a chemical stimulus, the flowmeters in the control box regulate the mix of medical air and CO_2_ to a specified concentration and flow rate. The manual/automated inputs provided to create a stimulus include the flow rate (mL/min), nominal CO_2_ concentration (%; 0% in case of mechanical and cold stimulus) and duration of the stimulus (seconds). The temperature of the stimulus is maintained throughout the experiment at either 50°C (translating to approximately 33°C at the ocular surface for mechanical and chemical stimulation) or room temperature for the cold stimulus. The nominal concentration is the %CO_2_ set by the observer/software for a given flow rate that would occur at the tip of the esthesiometer when the stimulus is presented.

### Carbon Dioxide Sensor

**Figure 3:**
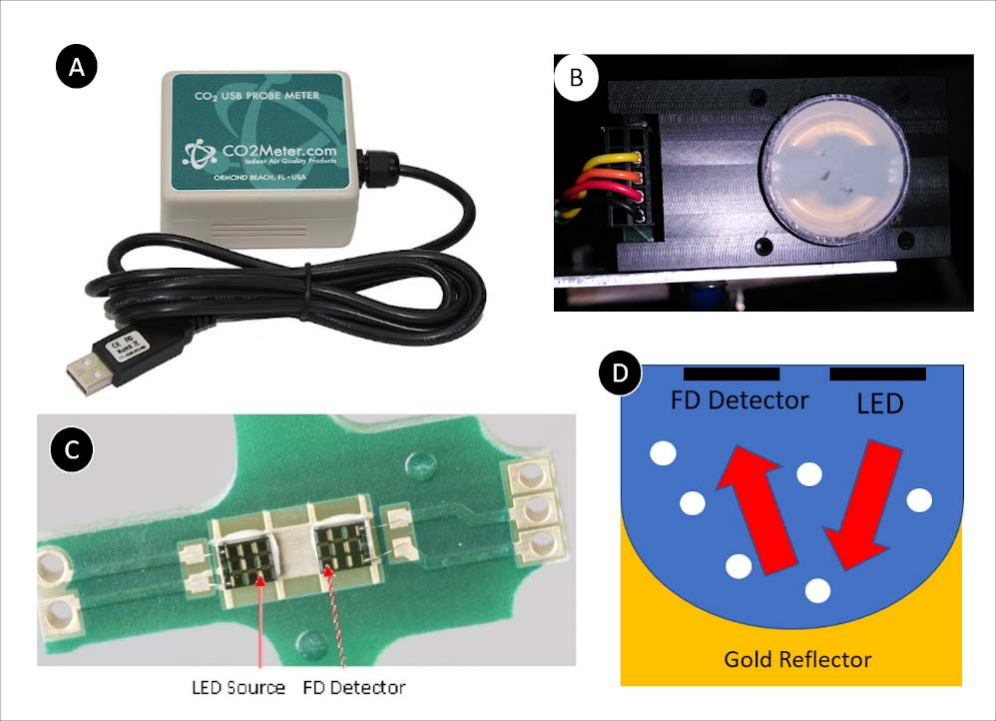
(A) COZIR CM-0041 portable carbon dioxide sensor^44^; (B) CO_2_ sensor without the protective case; (C) the bridgeboard containing LED source and detector to measure CO_2_ concentration (reference 43, included using creative commons attribution license 4.0); and (D) schematic representation of the measurement chamber of the CO_2_ sensor and CO_2_ detection mechanism (source: adapted from reference 43 using creative commons attribution license 4.0).

A portable CO_2_ sensor (COZIR CM-0041) from CO2Meter.com was used (Fig. 3).^45^ (According to the manufacturer, the CM-0041 has been discontinued. The GC-0016^45^ is the recommended replacement for the CM-0041, as both the models use the same COZIR 100% CO_2_ sensor.) This compact, low power, diffusion sampling sensor uses NDIR technology with gold-plated optics to measure ambient CO_2_ concentration. The measurement chamber is covered by a 100% CO_2_-permeable membrane for the CO_2_ molecules to enter the chamber. The information reviewed before choosing this particular type of sensor were its accuracy, sampling rate, optimal operating condition, and the ability to detect concentration from 0 to 100%. The COZIR CM-0041 sensor detects %CO_2_ from 0 to 100% with an accuracy of ±70 ppm or ±5% of the reading at a sampling rate of 2 Hz. Also, the optimal operating condition for this sensor was between 0 to 50°C/room temperature and atmospheric pressures between 950 mBar and 10 Bar. It could be used for an instantaneous measure of %CO_2_ or for fixed interval measure with the intermeasurement timing ranging from every second to every 30 minutes. The session data containing the time and concentration (ppm) could be exported to a spreadsheet by using the supplied data logger software. The sensor was precalibrated when purchased, and before each experimental session, the initial measurement of ambient room %CO_2_ was 1300 ± 100 ppm (average of 3 trials). In addition, as is reported later, when the stimulus was set to deliver 100% CO_2_ and the sensor was at the tip of the esthesiometer, it consistently reported 100% CO_2_.

### CO_2_ Sensor Design

The sensor design is as explained in the manufacturer's manual.^45^ The COZIR sensor uses an infrared LED light source and a detector (Fig. 3C)^43^ that is mounted on the bridgeboard facing the gold-plated parabolic reflector at the bottom (Fig. 3D). The active measurement area is the area between the bridgeboard and reflector. The LED is operated at 4.3 μm, as this wavelength is similar to the absorption spectra of CO_2_. The infrared light from the LED passes through the gas in the active area and reflects back to the detector by the reflector. The amount of light reaching the detector depends on the concentration of the CO_2_ inside the active area, and the rate of absorption or the proportion of light reaching the detector is used in the calculation of the %CO_2_ at a given moment.

### Experimental Setup

The sensor was removed from its original plastic enclosure (Fig. 3B) and mounted on the chin rest by using metal clamps for easier positioning of the sensor orthogonal to the tip of the esthesiometer (Fig. 1A). A calibrated measuring scale was used to adjust the working distance between the tip of the esthesiometer and front face of the sensor (Fig. 1B). The air vents in the room were partially blocked, and the room doors were closed to avoid air draft affecting the flow of the jet between the tip and sensor. The room setup was similar to the experiments with human participants performed in the lab. Due to the stimulus dimension and low flow rates used in the experiment, the stimulus duration for the trials was 99.9 seconds each (maximum time of the device) allowing the active area to get saturated with the stimulus being presented. The working distance, flow rate, and nominal concentrations were changed systematically according to the experiment. The %CO_2_ inside the active area of the sensor was logged every second. Between each trial, a breathing time of 2 minutes was used to allow the concentrations inside the chamber to return to ambient conditions. Each trial was done three times to measure repeatability. The concentration was measured at room temperature.

#### Experiment 1: Identifying an Optimal Location on the Surface of the Sensor to Deliver Corneal Pneumatic Stimuli

**Figure 4:**
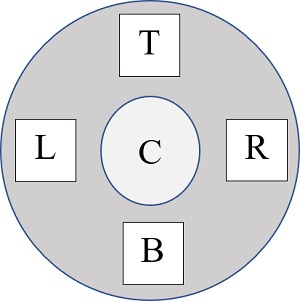
Location of area tested on the surface of the sensor indicated with the labels: C, center; L, left; R, right; T, top; B, bottom.

The diameter of the front face of the sensor is larger than the esthesiometer tip (stimulus column) as well as the active measurement area inside the sensor. Because the sensor is designed to detect ambient conditions, it was unclear what effect it would have on the detection of the %CO_2_ in the stimulus column. As seen in Figure 3B, the front face of the sensor with a bridgeboard (black shadow in the middle) obscures the entry of the stimulus into the sensor. Therefore, the tip of the esthesiometer was placed at five different locations on the surface of the collector (no loss in the CO_2_), and a stimulus of 100% CO_2_ at 100 mL/min flow rate was delivered directly to the active area. The locations were center, left, right, top, and bottom half of the sensor surface (Fig. 4).

#### Experiment 2: Effect of Flow Rate and Working Distance for a Maximum Nominal CO_2_ Concentration

This experiment was conducted to determine the concentration at the ocular surface plane with a constant stimulus concentration of 100%, and the flow rate varied at 3-, 5-, and 10-mm working distances. The flow rates used were 50, 100, 150, and 200 mL/min, and the flow rates were increased methodically from lowest to highest at each working distance.

#### Experiment 3: Estimation of %CO_2_ Reaching the Ocular Surface at Smaller Intervals of Flow Rate and Concentrations

In this experiment, all three components were changed to obtain their respective observed %CO_2_. The flow rate and concentration were varied in smaller steps at three predetermined working distances similar to experiment 2. The flow rates used were between 20 mL/min and 100 mL/min in 20-mL/min steps, whereas the concentrations were from 0 to 100% in 20% steps.

### Data Analysis

The maximum concentration achieved within each trial was extracted and used in the analysis. The data were analyzed using R statistics (version 3.4.3)^46^ in R studio (version 1.1.383)^47^. Linear models were obtained using “lme4,”^43^ and the test-retest repeatability was obtained using “irr” package.^48^,^49^ The plots were produced using “ggplot2”^50^and “cowplot”^51^ packages of R statistics.

## Results

### Determining the Feasibility and the Location of Stimulus Delivery

The feasibility was evaluated by delivering a 100% CO_2_ stimulus at a flow rate of 100 mL/min directly to the surface of the sensor. When delivered, the stimuli could still fill the active area with 100% CO_2_ when the tip was orthogonally positioned right against the surface in the top and bottom quadrants of the sensor. Even though the diameter of the esthesiometer tip/stimulus was smaller than the diameter of the collector, the stimulus could still saturate the chamber with 100% CO_2_, validating the use of the sensor in calibration. At locations other than the top and bottom quadrants, the observed %CO_2_ reached only 30% for a 100% CO_2_ stimulus, indicating a larger loss in the CO_2_ reaching the active area. As discussed in the construction of the sensor, the presence of bridgeboard may have (where the photo diode detector and LED are located) restricted/limited the CO_2_ molecules from entering the chamber, resulting in lower observed concentration. Because the CO_2_ molecules tend to rise when released, the stimuli for the experiments were delivered to the bottom half of the sensor for natural circulation of CO_2_ inside the active area of the sensor.

### Determining the Observed CO_2_

**Figure 5:**
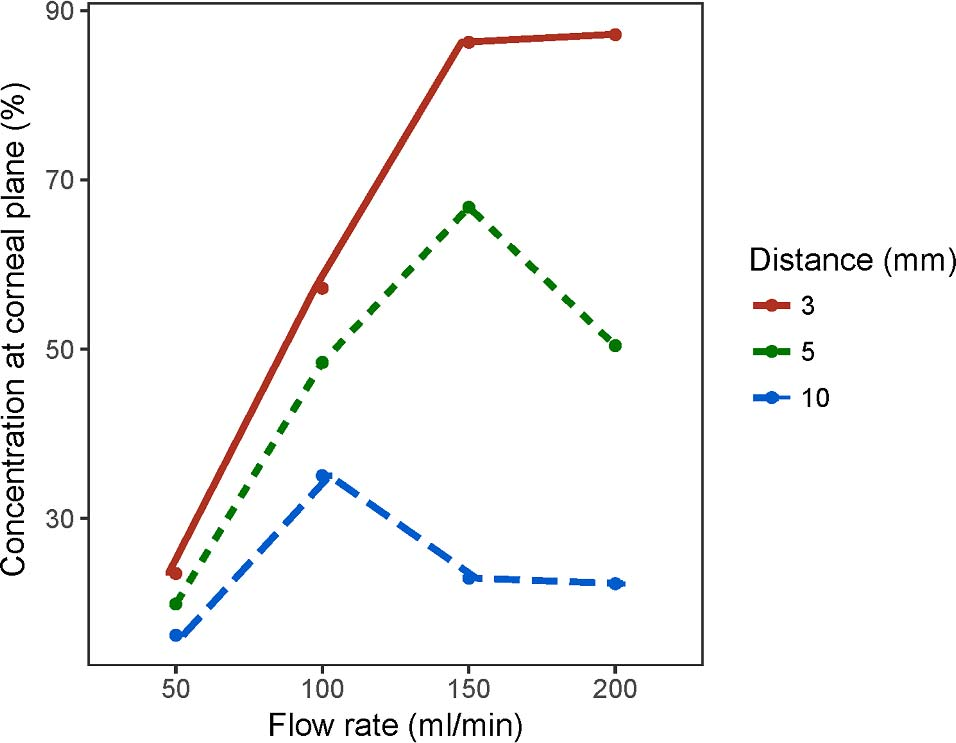
The observed concentration was plotted against the flow rate of the stimuli delivered with a concentration of 100% CO_2_. The colored lines indicate the working distance (distance between the sensor and esthesiometer tip) used in the trial.

**Figure 6:**
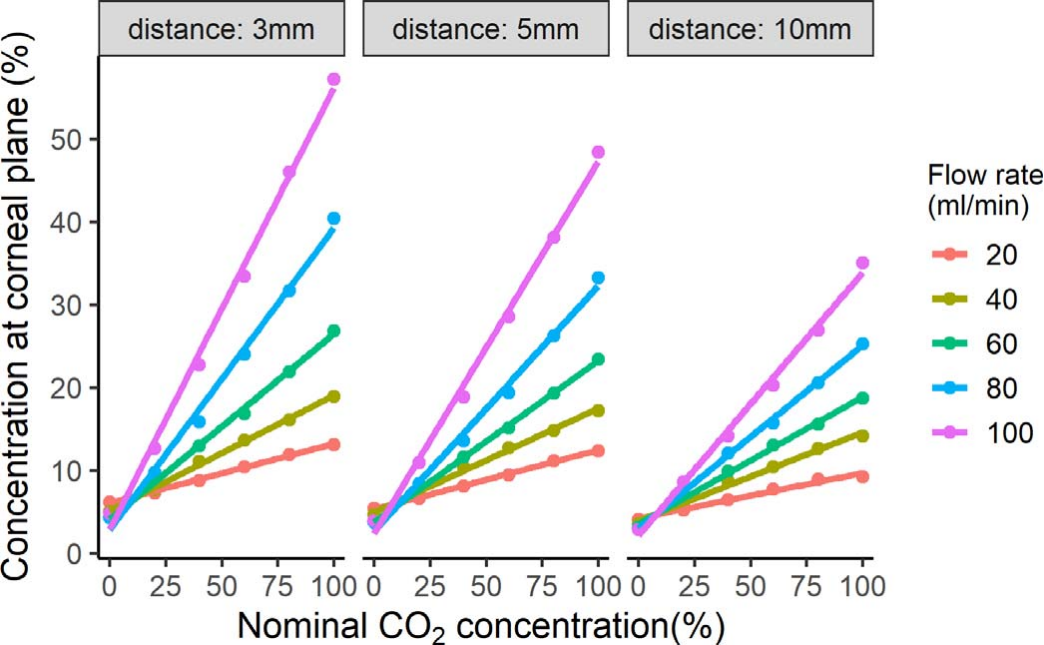
The observed concentration was plotted against nominal concentration. The linear fits were plotted for each working distance and flow rate.

The experiment with a fixed concentration (100% CO_2_) and a variable flow rate showed a progressive increase in the observed %CO_2_ with increasing flow rates, but the observed %CO_2_ values were relatively low at larger working distances compared to a 3-mm working distance (Fig. 5). The %CO_2_ values were strongest when the sensor was positioned 3 mm away from the tip, whereas the lowest was observed at 10 mm. A maximum concentration of 87.2% was obtained for a stimulus with the flow rate of 200 mL/min at 3 mm. Compared to low flow rates that had a linear increase in the %CO_2_, the amount of CO_2_ reaching the active measurement area lessened or plateaued at the strongest flow rates (150 and 200 mL/min) of the esthesiometer. In the subsequent experiment with the concentrations measured for flow rates within the usual test range and nominal %CO_2_ set at smaller steps, the rate of increase in the observed %CO_2_ corresponding to the nominal %CO_2_ was lower when the flow rates were lower and the sensor was positioned farther from the esthesiometer tip. There was a progressive increase in the variability of the observed %CO_2_ between flow rates with increasing nominal concentration resulting in a fan-like distribution of values at each working distance (Fig. 6). Both flow rate of the stimulus and working distance were found to be significantly important factors (*P* < 0.001) to determine the observed %CO_2_ reaching the ocular surface/sensor. Because the test-retest repeatability of each stimulus intensity was high with zero or small standard deviations for each mean (intraclass correlation coefficient [ICC] = 1), a nomogram was created using the average values so that the %CO_2_ at the ocular surface plane could be obtained based on the nominal concentration, working distance, and flow rate (Table 1).

**Table 1:** Nomogram to Obtain Observed Concentration at the Ocular Surface Plane for a Given Nominal Concentration, Flow Rate, and Working Distance

Flow Rate (ml/min)	Distance (mm)	Nominal %CO_2_				
		20	40	60	80	100
20	3	7.32	7.64	8.44	9.77	12.66
	5	6.62	7.23	7.61	8.39	10.93
	10	5.18	5.96	6.78	7.52	8.67
40	3	8.80	11.06	13.00	15.88	22.75
	5	8.10	10.44	11.60	13.58	18.88
	10	6.51	8.70	9.92	12.07	14.15
60	3	10.45	13.64	16.85	24.02	33.37
	5	9.46	12.71	15.10	19.40	28.58
	10	7.75	10.45	13.03	15.73	20.30
80	3	11.91	16.10	21.93	31.73	46.02
	5	11.21	14.86	19.29	26.22	38.11
	10	8.94	12.65	15.60	20.58	26.88
100	3	13.09	18.94	26.87	40.41	57.02
	5	12.38	17.23	23.40	33.22	48.43
	10	9.20	14.14	18.67	25.27	35.05

## Discussion

Pneumatic esthesiometry is currently the only way to examine chemonociception on the human ocular surface. These experiments are more time-consuming than other pneumatic esthesiometry experiments because of the necessity to remove the gas from the previous trials. There is currently only one esthesiometer specifically designed with a vacuum component to do this without slowing down the experiments,^52^ and its CO_2_ characteristics have also not been experimentally determined. In this study, we examined the feasibility of using a relatively inexpensive portable CO_2_ sensor to calibrate the chemical (CO_2_) stimuli of our pneumatic esthesiometer at the ocular surface plane. The research question arose because, perhaps, the CO_2_ stimuli were internally calibrated, and the composition of the stimulus is unknown when it reaches the ocular surface. Because the stimulus released from the esthesiometer interacts with the environment before reaching the area of interest, calibrating the stimulus at the ocular surface would help in improving the experimental design to measure chemical sensitivity.

### Feasibility

The feasibility was primarily tested because the column of gas produced by the esthesiometer was limited (diameter at the nozzle tip is 0.5 mm) and in our esthesiometer, the stimulus column (from the nozzle tip to the ocular surface) was 5 mm long, whereas the front face of the collector was 20 mm in diameter. It was unclear that the CO_2_ measuring device would be able to reliably detect/measure the gas within the limits of the gas column and, in addition, if it were able to, what would be the characteristics of the column (or at least the characteristics of CO_2_ within the column) determined by the sensor. Calibration of the esthesiometer using the sensor seemed generally viable based on the results obtained for both medical air (0% CO_2_) and 100% CO_2_ stimuli. The CO_2_ measurements were accurate (based on readings with zero added and 100% CO_2_ columns) and repeatable, even though there was a mismatch between the sensor output and nominal stimulus specifications.

### Relationship between Concentration, Flow Rate, and Working Distance

A linear relationship was observed between nominal and observed concentrations for flow rates up to 100 mL/min depending on the working distance (Figs. 5 and 6). There was a reduction in the observed %CO_2_ at high flow rates (Fig. 5), which might be due to the turbulence in the stimulus or disruption in the laminar flow of the stimulus allowing the CO_2_ to diffuse out of the stimulus column. The decrease in the %CO_2_ was more pronounced when the sensor was placed away from the esthesiometer. Of course, the interaction between the stimulus air-column and surrounding air is to be expected and has been shown using Schlieren imaging of the mechanical stimulus, which showed turbulence fringes at higher flow rates.^10^ From our perspective when using pneumatic esthesiometry to measure psychophysical sensory performance, fortunately, the flow rate for chemoreception trials would never be more than 100 mL/min in our experiments. To minimize the mechanical sensory effect while measuring chemical thresholds, the flow rate of the stimulus would always be set at half of the mechanical (flow) thresholds and the maximum flow rate for the Waterloo modified esthesiometer is 200 mL/min. Although there were suggestions that the relationships can be more complicated with some nonlinearities at higher flow rates, both of these attributes (the general “simple relationships” as well as the departure from what is expected) highlight the importance of understanding how the air column behaves to understand the sensory attributes of the tissue being examined when doing pneumatic esthesiometry, in our instance, of the ocular surface.

#### Repeatability

The study by Tesón et al.^31^ found that the chemical thresholds were the least repeatable thresholds among the corneal sensory measurements, with a variability of 18.06% and ICC of 0.49. This is the only study that measured repeatability of the chemical threshold. Many internal and external factors have been found to vary the ocular surface sensitivity.^53^ Calibration could be a factor that is closely related to the stimulus characteristics for the variability in the sensation perceived by the participants. In our study, we found that the chemical stimuli itself is repeatable (ICC = 1) considering the flow rate and working distance remain constant between the trials. When the flow rates were increased, there was an increase in the observed concentration at the ocular surface plane even though the concentration delivered remained same (Fig. 5). This phenomenon was easily noticeable at higher nominal concentrations, and a similar phenomenon was observed with the working distance that was discussed earlier. In a human ocular sensitivity experiment, the working distance will remain constant among all participants, but the flow rate is different between participants based on the mechanical thresholds. There might be a confounding factor in the form of flow rates that may reduce the repeatability of the chemical thresholds in human participants. The information on the difference in the flow rate of chemical stimuli were not available in the study by Tesón et al.^31^

#### Complexity of Regression Models

**Figure 7:**
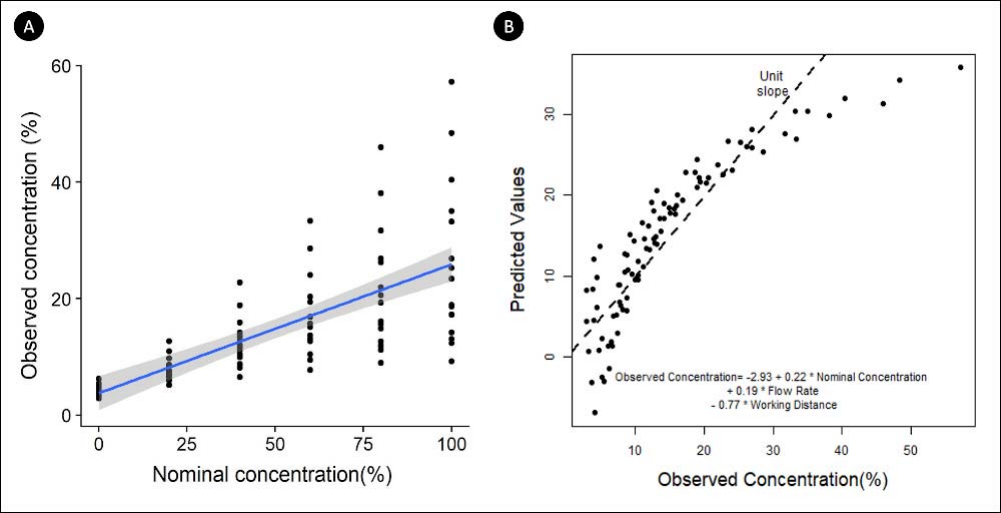
(A) The observed concentrations were plotted against the nominal concentrations and a linear fit was added with flow rate and working distance as factors. (B) The predicted values were plotted against the observed concentrations in the scatter plot. The %CO_2_ was predicted using the linear equation annotated in the figure and compared with the observed concentration from the sensor. Ideally, all points would be on the y = x function (*dotted line)*.

One of the aims of this study was to create a regression model that predicts the observed concentration based on the nominal concentration, flow rate, and working distance. Mixed modeling and nonlinear multiple regression models were attempted to create the expected models. Partly due to very poorly behaved error distributions and fan-like distribution of observed values, the predictability of the models was poor especially at higher concentrations (Fig. 7). Because of the residual errors, simple linear regression lines were fitted to data for each flow rate at each working distance. The r-square values for all the linear regression lines were more than 0.95, indicating good fit (Table 2).

**Table 2:** Linear Regression Equations to Calculate Observed Concentration Based on Flow Rate, Working Distance, and Nominal Concentration (x)

Flow Rate (ml/min)	3 mm		5 mm		10 mm	
	Equation	*r*^2^	Equation	*r*^2^	Equation	*r*^2^
20	6.07 + 0.073x	0.997	5.30 + 0.071x	0.998	4.20 + 0.055x	0.977
40	5.12 + 0.139x	0.998	4.93 + 0.125x	0.996	3.91 + 0.107x	0.992
60	4.07 + 0.224x	0.998	3.75 + 0.195x	0.999	3.51 + 0.153x	0.997
80	2.84 + 0.364x	0.993	2.69 + 0.295x	0.993	2.99 + 0.221x	0.999
100	2.87 + 0.532x	0.995	2.40 + 0.448x	0.995	2.16 + 0.317x	0.995

#### Limitations

Even though we were able to calibrate the chemical stimuli, there is still an inability to measure the %CO_2_ in the stimulus column instantly, and the temperature of the stimulus was also not same as the one used in a regular experiment. The inability for an instantaneous measure of concentration in the stimulus column might be due to the size of the measurement chamber and the diffusion model of the sensor. In this study, we overcame the limitation by delivering the stimulus for an extended period to saturate the measurement chamber with the stimulus presented, and the maximum concentration attained within the trial was used as the observed concentration. The saturation of the gas inside the chamber can be monitored in the real-time graph of the data logger software provided. As soon as the chemical stimulus was on, the observed %CO_2_ increased sharply from the baseline (ambient level) until it plateaued or slowed the increase in the concentration with time. The plateauing was apparent at high flow rates and closer working distance. The plateau indicated the saturation of the %CO_2_ inside the chamber and there was no evidence of CO_2_ pooling inside the chamber, as the %CO_2_ started dropping instantaneously after the stimulus was off. The %CO_2_ inside the chamber returned to ambient levels within half a minute after the stimulus was off.

In this study, the temperature of the stimulus was not the same as the one for human ocular surface chemoreception experiments because the NDIR sensor used in this experiment uses infrared LED to detect the concentration. A change in the temperature of the stimulus might affect the performance of the sensor as well as the temperature of the stimuli would, themselves, require additional calibrations. The 50°C temperature at the nozzle is designed explicitly for a stimulus delivered from a 5-mm working distance (as it translates to 33°C or normal ocular surface temperature when it reaches the ocular surface) and it may not translate to the ocular surface temperature at other working distances. As this calibration study explored the effects (among others) of other working distances to characterize the CO_2_ in the stimulus, altering the thermal gradient would alter the sensor performance.

The sensor setup in this manuscript does not exactly reflect a human ocular surface experiment. The facial features such as nose and deep eye socket might provide a more closed environment affecting the air circulation and altering the dispersion of the CO_2_ from and surrounding air into the stimulus air column. In addition, body temperature and the thermal gradient surrounding the body might be expected to influence these flows, in addition to the physical structure of facial features. We have previously shown that blocking the flow from and into the column by using a tube (obviously) does affect the distribution of measured CO_2_^54^; there was an increase in the CO_2_ reaching the sensor. This might better control the concentration of the CO_2_, but it cannot be implemented clinically because of the effect of the tube on the cornea and eyelids. Future calibrations more accurately simulating the ocular surface environment (including different brow and nose characteristics and eye socket depths and ocular temperature) would provide information about the influences that these theoretical variables would have over the stimulus air column.

### Recommendations

We would like to suggest a 5-mm working distance for ocular surface sensory processing experiments with the pneumatic esthesiometer. This particular working distance is recommended because the 3-mm working distance is too close to the eye and the esthesiometer tip will touch the eyelid/lashes, producing discomfort and false responses from the participants. On the other hand, longer working distances have the primary disadvantage of not being able to provide sufficient CO_2_ concentrations at the eye to enable consistent measurements of thresholds, and other experimentation also requires higher amounts of CO_2_ delivery, e.g., adaptation experiments.^55^,^56^

### Summary

Calibration of the CO_2_ in the air column of a pneumatic esthesiometer is critical. There is a systematic reduction in the %CO_2_ reaching the ocular surface plane that depends on working distance and flow rate. The measures of CO_2_ were repeatable for all stimulus combinations. It is evident that in pneumatic esthesiometers, it is necessary to standardize the chemical stimulus, as both working distance and flow rate could change the amount of CO_2_ reaching the ocular surface.

## References

[1] Belmonte C, Giraldez F (1981). Responses of cat corneal sensory receptors to mechanical and thermal stimulation. *J Physiol*.

[2] Boberg-Ans J (1955). Experience in clinical examination of corneal sensitivity. *Br J Ophthalmol*.

[3] Lele PP, Weddell G (1956). The relationship between neurohistology and corneal sensibility. *Brain*.

[4] Cochet P, Bonnet R (1960). L'esthésie cornéenne. *La Clin Ophthalmol*.

[5] Schirmer KE (1963). Corneal sensitivity and contact lenses. *Br J Ophthalmol*.

[6] Lele PP, Weddell G (1959). Sensory nerves of the cornea and cutaneous sensibility. *Exp Neurol*.

[7] Beuerman RW, Tanelian DL (1979). Corneal pain evoked by thermal stimulation. *Pain*.

[8] Gallar J, Pozo MA, Tuckett RP, Belmonte C (1993). Response of sensory units with unmyelinated fibres to mechanical, thermal and chemical stimulation of the cat's cornea. *J Physiol*.

[9] Chen X, Gallar J, Pozo MA, Baeza M, Belmonte C (1995). CO2 stimulation of the cornea: a comparison between human sensation and nerve activity in polymodal nociceptive afferents of the cat. *Eur J Neurosci*.

[10] Murphy PJ, Patel S, Marshall J (1996). A new non-contact corneal aesthesiometer (NCCA). *Ophthalmic Physiol Opt*.

[11] Vega JA, Simpson TL, Fonn D (1999). A noncontact pneumatic esthesiometer for measurement of ocular sensitivity: a preliminary report. *Cornea*.

[12] Von Frey M (1894). Beitrage zur physiologie des schmerzes (Contribution to the Physiology of Pain).

[13] Draeger J (1984). Corneal Sensitivity: Measurement and Clinical Importance. Springer Science and Business Media.

[14] Schirmer KE (1963). Assessment of corneal sensitivity. *Br J Ophthalmol*.

[15] Larson WL (1970). Electro-mechanical corneal aesthesiometer. *Br J Ophthalmol*.

[16] Millodot M (1984). A review of research on the sensitivity of the cornea. *Ophthalmic Physiol Opt*.

[17] Golebiowski B, Papas E, Stapleton F (2011). Assessing the sensory function of the ocular surface: Implications of use of a non-contact air jet aesthesiometer versus the Cochet-Bonnet aesthesiometer. *Exp Eye Res*.

[18] Millodot M, Larson WL (1967). Effect of bending the nylon thread of the Cochet Bonnet aesthesiometer upon the recorded pressure. *Contact Lens*.

[19] Brennan NA, Maurice DM (1989). Corneal esthesiometry with a carbon dioxide laser. *Investig Ophthalmol Vis Sci*.

[20] Belmonte C, Gallar J, Pozo MA, Rebollo I (1991). Excitation by irritant chemical substances of sensory afferent units in the cat's cornea. *J Physiol*.

[21] Pozo MA, Gallego R, Gallar J, Belmonte C (1992). Blockade by calcium antagonists of chemical excitation and sensitization of polymodal nociceptors in the cat's cornea. *J Physiol*.

[22] Gonzalez GG, De la Rubia PG, Gallar J, Belmonte C (1993). Reduction of capsaicin-induced ocular pain and neurogenic inflammation by calcium antagonists. *Investig Ophthalmol Vis Sci*.

[23] Gonzalez GG, Gallar J, Belmonte C (1995). Influence of diltiazem on the ocular irritative response to nitrogen mustard. *Exp Eye Res*.

[24] Belmonte C, Acosta MC, Schmelz M, Gallar J (1999). Measurement of corneal sensitivity to mechanical and chemical stimulation with a CO2 esthesiometer. *Investig Ophthalmol Vis Sci*.

[25] Feng Y, Simpson TL (2003). Nociceptive sensation and sensitivity evoked from human cornea and conjunctiva stimulated by CO2. *Investig Ophthalmol Vis Sci*.

[26] Situ P, Simpson TL, Fonn D (2007). Eccentric variation of corneal sensitivity to pneumatic stimulation at different temperatures and with CO2. *Exp Eye Res*.

[27] Acosta M, Tan M, Belmonte C, Gallar J (2001). Sensations evoked by selective mechanical, chemical, and thermal stimulation of the conjunctiva and cornea. *Investig Ophthalmol Vis Sci*.

[28] De Paiva CS, Pflugfelder SC (2004). Corneal epitheliopathy of dry eye induces hyperesthesia to mechanical air jet stimulation. *Am J Ophthalmol*.

[29] Feng Y, Simpson TL (2004). Characteristics of human corneal psychophysical channels. *Investig Ophthalmol Vis Sci*.

[30] Acosta M, Belmonte C, Gallar J (2001). Sensory experiences in humans and single-unit activity in cats evoked by polymodal stimulation of the cornea. *J Physiol*.

[31] Tesón M, Calonge M, Fernández I, Stern ME, González-García MJ (2012). Characterization by belmonte's gas esthesiometer of mechanical, chemical, and thermal corneal sensitivity thresholds in a normal population. *Investig Ophthalmol Vis Sci*.

[32] Belmonte C, Acosta MC, Gallar J (2004). Neural basis of sensation in intact and injured corneas. *Exp Eye Res*.

[33] Belmonte C, Garcia-Hirschfeld J, Gallar J (1997). Neurobiology of ocular pain. *Prog Retin Eye Res*.

[34] Spierer O, Felix ER, McClellan AL (2016). Corneal mechanical thresholds negatively associate with dry eye and ocular pain symptoms. *Investig Ophthalmol Vis Sci*.

[35] Wu Z, Begley CG, Port N, Bradley A, Braun R, King-Smith E (2015). The effects of increasing ocular surface stimulation on blinking and tear secretion. *Investig Ophthalmol Vis Sci*.

[36] Situ P, Simpson TL, Begley CG (2015). Test-retest repeatability and calibration of a new automated Belmonte esthesiometer. *Investig Ophthalmol Vis Sci*.

[37] Stapleton F, Tan ME, Papas EB (2004). Corneal and conjunctival sensitivity to air stimuli. *Br J Ophthalmol*.

[38] Murphy PJ, Lawrenson JG, Patel S, Marshall J (1998). Reliability of the non-contact corneal aesthesiometer and its comparison with the Cochet-Bonnet aesthesiometer. *Ophthalmic Physiol Opt*.

[39] Nosch DS, Pult H, Albon J, Purslow C, Murphy PJ (2016). Relationship between corneal sensation, blinking, and tear film quality. *Optom Vis Sci*.

[40] Efron N, Young G, Brennan NA (1989). Ocular surface temperature. *Curr Eye Res*.

[41] Zhou R, Vaihinger S, Geckeler KE, Göpel W (1994). Reliable CO2 sensors with silicon-based polymers on quartz microbalance transducers. *Sensors Actuators B Chem*.

[42] Nosch DS, Pult H, Albon J, Purslow C, Murphy PJ (2018). Does air gas aesthesiometry generate a true mechanical stimulus for corneal sensitivity measurement?. *Clin Exp Optom*.

[43] Gibson D, MacGregor C (2013). A novel solid state non-dispersive infrared CO2 gas sensor compatible with wireless and portable deployment. *Sensors*.

[44] Situ P (2010). Sensitivity across the ocular surface—fundamental findings and clinical applications.

[45] CO2Meters.com DataSheet: COZIR.

[46] R Core Team (2008). R: a language and environment for statistical computing. R Foundation for Statistical Computing.

[47] (2016). Rstudio. Integrated development environment for R.

[48] Bates D, Mächler M, Bolker B, Walker S (2015). Fitting linear mixed-effects models using {lme4}. *J Stat Softw*.

[49] Duforet-Frebourg N, Luu K, Laval G, Bazin E, Blum MGB (2016). Detecting genomic signatures of natural selection with principal component analysis: application to the 1000 genomes data. *Mol Biol Evol*.

[50] Wickham H (2009). *ggplot2: Elegant Graphics for Data Analysis*.

[51] Wilke CO (2017). cowplot: streamlined plot theme and plot annotations for “ggplot2.”.

[52] Situ P, Simpson T, Begley C (2016). Hypersensitivity to cold stimuli in symptomatic contact lens wearers. *Optom Vis Sci*.

[53] Golebiowski B, Papas EB, Stapleton F (2008). Factors affecting corneal and conjunctival sensitivity measurement. *Optom Vis Sci*.

[54] Jayakumar V, Alabi E, Feng Y, Simpson TL (2015). Calibration of chemical pneumatic Belmonte esthesiometer stimuli using a portable carbon dioxide sensor. *Invest Ophthalmol Vis Sci*.

[55] Chen J, Simpson TL (2011). A role of corneal mechanical adaptation in contact lens-related dry eye symptoms. *Investig Ophthalmol Vis Sci*.

[56] Chen J, Feng Y, Simpson TL (2010). Human corneal adaptation to mechanical, cooling, and chemical stimuli. *Investig Ophthalmol Vis Sci*.

